# Assessing nurses’ attitudes toward artificial intelligence in Kazakhstan: psychometric validation of a nine-item scale

**DOI:** 10.3389/fdgth.2026.1756338

**Published:** 2026-04-01

**Authors:** Shnara Svetlanova, Balnur Iskakova, Dinara Makhanbetkulova, Aurelija Blazeviciene, Laila Nazarova, Nadira Aitambayeva, Nazerke Narymbayeva, Bibinur Sydykova, Tamara Abdirova, Ainur Qumar

**Affiliations:** 1 Department of Health Policy and Management, Asfendiyarov Kazakh National Medical University, Almaty, Kazakhstan; 2 Kazakhstan Medical University “KSPH”, Almaty, Kazakhstan; 3 Department of Nursing, Asfendiyarov Kazakh National Medical University, Almaty, Kazakhstan; 4 Department of Nursing, Faculty of Nursing, Medical Academy, Lithuanian University of Health Sciences, Kaunas, Lithuania

**Keywords:** artificial intelligence, attitudes, Kazakhstan, nurses, psychometric validation

## Abstract

**Background:**

Artificial intelligence (AI) is increasingly integrated into healthcare, yet the attitudes and knowledge of nurses, who are the key mediators of AI implementation, remain underexplored. This study aimed to evaluate the psychometric properties of a previously validated nine-item scale measuring nurses’ knowledge and attitudes toward AI and to describe preliminary findings from primary healthcare centre (PHC) nurses in Almaty, Kazakhstan.

**Methods:**

A cross-sectional survey was conducted among 400 nurses from eight randomly selected PHCs in Almaty. The English version of the questionnaire assessing sociodemographic characteristics, knowledge of AI, and attitudes toward AI among nurses was translated and adapted in Kazakh and Russian languages. Exploratory factor analysis (EFA) was performed on 60% of the sample (*n* = 240) to identify the factor structure, followed by confirmatory factor analysis (CFA) on the remaining 40% (*n* = 160). Internal consistency, composite reliability, and average variance extracted were calculated to evaluate reliability and convergent validity.

**Results:**

Most participants were female (94%, *n* = 376), aged 20–39 years (51.5%,*n* = 206), and held post-secondary medical college education (52.5%,*n* = 210). About one third of the participants reported having no general awareness of AI, and nearly half (45.8%, *n* = 183) reported little to no understanding of the use of AI in nursing. EFA supported a two-factor structure “Operational and Workforce Impact” and “Clinical Benefits” explaining 72.7% of the variance. CFA confirmed the model with good model fit indices and high internal consistency (Cronbach's *α* overall=0.94; subscales 0.92 and 0.89). A substantial proportion of nurses recognized AI's potential to enhance patient care, decision-making, and workflow efficiency, though 38.8% were reluctant to adopt AI personally.

**Conclusions:**

The validated scale demonstrated excellent psychometric properties in Kazakhstani context and has the potential to be used more broadly across the Central Asian region. While nurses exhibited positive perceptions of AI's clinical and operational benefits, gaps in specific knowledge suggest a need for targeted educational interventions.

## Introduction

The concept of artificial intelligence (AI) originated in the 1950s, largely inspired by Alan Turing's pioneering ideas on machine intelligence (1). Since then, AI has evolved into a key driver of digital transformation, characterised by systems capable of interpreting data, learning from experience, and autonomously performing complex tasks. Over recent decades, AI technologies have been increasingly integrated into diverse industries, including healthcare, where they are now viewed as essential tools for advancing clinical practice and improving health outcomes (1, 2).

In the medical field, AI applications range from diagnostic support systems and predictive analytics to the development of personalised treatment strategies. By enhancing the accuracy of diagnosis, optimising treatment planning, and enabling real-time patient monitoring, AI has the potential to significantly improve the quality and efficiency of healthcare delivery (3, 4). For example, AI-driven systems can analyse data from wearable devices to detect early signs of patient deterioration, allowing nurses and clinicians to intervene promptly and effectively (4). Moreover, predictive analytics can support workforce management by optimising staff scheduling and resource allocation, thereby reducing routine workloads and improving operational efficiency within healthcare institutions (5).

Within the nursing profession, AI has become a particularly powerful tool for strengthening clinical decision-making, workflow efficiency, and patient outcomes. As frontline providers, nurses play a central role in the implementation and daily use of AI systems in clinical settings, mediating their integration into patient care processes (6). AI-based decision support tools can synthesise large amounts of patient information, identify risk factors, and generate evidence-based recommendations, thereby enhancing nurses’ critical thinking and diagnostic reasoning (7–9). Furthermore, automation supported by AI reduces documentation and administrative burden, potentially allowing nurses to devote more time to direct patient care (9, 10). AI technologies can also optimise workload distribution by aligning nursing tasks with patient acuity and staff competencies, helping reduce burnout and promote high-quality care delivery (11).

AI is rapidly emerging as a transformative force within Kazakhstan's healthcare system too. The government has shown growing support for digital health initiatives, including AI-driven solutions, through national strategies aimed at modernising healthcare infrastructure, promoting telemedicine, and integrating smart technologies into clinical practice (12). Previous pilot programs in AI-based remote patient monitoring have demonstrated reduction in hospital admissions and a decrease in treatment costs for patients with chronic conditions (13). Other initiatives include AI-assisted diagnostic platforms in urban hospitals and predictive analytics tools for managing patient flows, both of which have begun to improve care efficiency and resource allocation (14). Public perception studies indicate that patients generally view these technologies positively, appreciating their potential to enhance care, while emphasising the need for human oversight, data privacy, and transparency (15). However, regulatory frameworks specific to healthcare AI remain underdeveloped, prompting calls for contextualised policies to ensure safe, ethical, and equitable implementation (14, 16).

Amid these ambitious interventions, the roles of medical staff, including nurses, often remain underexplored. Assessing nurses’ attitudes toward AI is therefore essential for anticipating implementation barriers and informing workforce preparedness strategies. Equally important is the use of standardized, culturally adapted measurement tools, as these ensure the validity, reliability, and comparability of responses across settings. In recent years, several instruments have been developed to assess healthcare professionals’ attitudes, knowledge, and readiness toward AI, with many demonstrating good psychometric performance and increasing methodological refinement (17–20). Nevertheless, most of them were developed within limited cultural and linguistic contexts, and there remains a notable scarcity of validated instruments available in Russian or Kazakh languages. This gap is particularly relevant given the rapid digitalisation of healthcare systems across post-Soviet countries (12, 13). This study aimed to evaluate the psychometric properties of a previously validated nine-item scale on nurses’ knowledge and attitudes toward AI in Kazakhstani context. Nurses in Kazakhstan frequently experience substantial administrative and clinical burden, making brief and easily deployable instruments especially valuable for ensuring adequate participation in research. Additionally, the study provides preliminary descriptive results of knowledge and attitudes towards AI among healthcare workers based on a representative sample of primary healthcare nurses in Almaty, Kazakhstan.

## Methods

### Study design and setting

Eight primary healthcare centres (PHC) were randomly selected from a sampling frame of 65 PHCs in Almaty. The research team first contacted the chief medical officers of the selected clinics to facilitate recruitment. These officers distributed the study invitations via WhatsApp groups that included all nurses in their respective clinics. Data were collected during the period of June-July in 2025. Participation was entirely voluntary, and clinic administration did not have access to information regarding who chose to complete the survey. Eligible participants were required to be at least 18 years old, fluent in Russian or Kazakh, and have a minimum of one year of work experience in healthcare. A total of 400 nurses provided informed consent and completed the online survey, which took approximately 15–20 min to complete.

### Measures and questionnaire

Data were collected using a structured questionnaire adapted from a previously published English version of the instrument (21). For each study language, one investigator first translated the questionnaire from English into the target language (Russian or Kazakh), and another investigator independently back-translated it into English. Discrepancies between the original and back-translated versions were then discussed and resolved within the research team to ensure both linguistic and conceptual equivalence (SS, BS worked on the Russian version while AQ, BI worked on the Kazakh version of the questionnaire). In addition, the translated versions were reviewed by a practicing nurse to assess item clarity, relevance, and appropriateness within the local clinical context. During this review, the practicing nurse did not suggest any major modifications, indicating that the items were generally clear, relevant, and appropriate for the local clinical context. Formal pilot testing of the questionnaire was not conducted prior to data collection. The final version of the questionnaire comprised three sections:

#### Sociodemographic and professional characteristics

Sociodemographic variables included age, sex, place of residence (urban or rural), highest educational attainment (post-secondary medical college, applied bachelor's degree, academic bachelor's degree, master's degree, or doctoral degree), and professional qualification category. In Kazakhstan, the professional qualification system for healthcare personnel follows a nationally standardized framework established by the Ministry of Health (22). The categories are: “No category” (entry-level or newly qualified specialists), “Second category” (clinicians with basic professional experience and competence), “First category” (professionals with advanced experience and demonstrated proficiency), and “Highest category” (professionals with extensive experience and leadership roles). For this study, we adopted this standardized classification for the professional qualification variable.

#### Knowledge of AI in healthcare

Knowledge on general AI and specifically in healthcare was assessed using two self-reported items adapted from the original instrument: “Do you have an understanding of artificial intelligence?” and “Do you have an understanding of the application of artificial intelligence in nursing?”. Each item was rated using a four-point response scale: “No, not at all,” “Understand a little,” “Understand mostly,” and “Understand fully.”

#### Attitudes toward AI

Attitudes towards AI in healthcare scale included nine items measuring perceived advantages and disadvantages of AI and willingness to use AI in nursing practice (Supplementary file 1). Example items included statements such as “Do you agree that that the application of artificial intelligence will reduce the burden on healthcare workers?” Participants rated their level of agreement on a 4-point Likert scale from “Strongly agree” to “Strongly disagree”.

The survey was administered online using Google Forms. Facility coordinators distributed the survey link to eligible nurses across the eight selected PHCs of Almaty. Survey completion was anonymous; no personal identifiers were collected. Required fields were used for core items to minimize missing data (23).

### Statistical analysis

Data analysis was conducted using R Studio software (version 4.3.0)**.** The raw dataset was initially prepared in Microsoft Excel 2021 and imported into R for analysis**.** Categorical variables were summarized using absolute numbers and percentages.

To examine the structure and psychometric properties of the questionnaire assessing attitudes toward AI in healthcare in Kazakh and in Russian, a two-step factor analysis approach was applied. The full sample was randomly divided into two independent subsamples to permit exploratory factor analysis (EFA) for factor identification and confirmatory factor analysis (CFA) for subsequent validation, thereby reducing the risk of overfitting. Although no fixed split ratio is mandated, the literature recommends partitioning a single dataset into two sufficiently large subsamples to ensure adequate statistical power for both exploratory and confirmatory analyses (24).

First, the EFA was performed on a randomly selected subset of approximately 60% of participants (*n* = 240) to identify the underlying factor structure of the AI perception scale. Prior to conducting the EFA, the Kaiser–Meyer–Olkin (KMO) measure of sampling adequacy and Bartlett's test of sphericity were applied to verify data suitability for factor analysis (24). A scree plot and parallel analysis were used to determine the optimal number of factors. Factor loadings of ≥0.35 were considered acceptable for item retention. Internal consistency of the identified factors was evaluated using Cronbach's alpha, with values above 0.60 considered acceptable.

Next, the CFA was carried out on the remaining 40% of the sample (*n* = 160)**,** which was independent of the EFA subset, to validate the two-factor model obtained from the exploratory analysis. CFA was conducted using the lavaan package (version 0.6–19) with maximum likelihood estimation. Model fit was assessed using several fit indices: the chi-square (*χ*^2^) test**,** Comparative Fit Index (CFI)**,** Tucker–Lewis Index (TLI)**,** Root Mean Square Error of Approximation (RMSEA) with 90% confidence interval (CI)**,** and Standardized Root Mean Square Residual (SRMR)**.** Acceptable model fit was indicated by CFI and TLI ≥0.90**,** RMSEA ≤ 0.08**,** and SRMR ≤ 0.08 (25).

Convergent validity was assessed using Composite Reliability (CR) and Average Variance Extracted (AVE)**,** with thresholds of CR > 0.70 and AVE > 0.50 considered indicative of satisfactory reliability and shared variance within each construct. Internal consistency reliability was further examined for both the overall scale and its subscales using Cronbach's alpha (*α*) coefficients. All statistical tests were two-tailed, and a significance level of *p* < 0.05 was used throughout.

Additionally, to explore associations between individual AI perception items and key sociodemographic factors, bivariate analyses using chi-square tests (*χ*^2^) were performed. For this purpose, all AI perception items and exposure variables were dichotomized: Likert-scale responses were converted to binary categories (e.g., “Agree” vs. “Disagree”), and sociodemographic variables with multiple categories, such as age and professional qualification, were also recoded into two groups (e.g., age <40 vs. ≥ 40; highest vs. lowest professional category). Chi-square tests (*χ*^2^) were conducted for each AI perception item separately against age, sex, professional category, and place of residence. Results are presented as *χ*^2^ statistics with corresponding *p*-values, and *p*-values <0.05 were considered statistically significant.

### Ethical considerations

Ethical approval for the current study was obtained from Asfendiyarov Kazakh National Medical University (No. 28/164 dated 27.05.2025). All participants were fully informed about the study aims and procedures prior to completing the online questionnaire. Written informed consent was obtained electronically before participation. No identifying information was collected, and participant anonymity and confidentiality were strictly maintained throughout the study, in accordance with institutional guidelines and international ethical standards.

## Results

### Socio-demographic characteristics

The socio-demographic characteristics of the sample are presented in Table 1. In this survey, women constituted the vast majority of participants (94.0%, *n* = 376). Such outstanding number of female participants can be related to the fact that healthcare sector itself is female dominated in the country. The most common age range of respondents was 20–39 years (51.5%, *n* = 206). More than two-thirds of participants (73.5%, *n* = 294) resided in urban areas. Over half of the respondents (52.5%, *n* = 210) reported holding a post-secondary medical college education, while a small proportion (1.5%, *n* = 6) had completed a master's program. Regarding professional qualification categories nearly half of the nurses (47.5%, *n* = 190) reported having no category and around one third had the highest category (31.8%, *n* = 127).

**Table 1:** Socio-demographic characteristics of the sample (*n*=400).

Variable	Category	N	%
Gender	Female	376	94
	Male	24	6
Age	Under 20 years	14	3.5
	20–39 years	206	51.5
	40–59 years	169	42.2
	60 years and older	11	2.8
Place of residence	Urban	294	73.5
	Rural	106	26.5
Education level	College level	210	52.5
	Applied bachelor	100	25
	Academic bachelor	84	21
	Master's degree	6	1.5
Professional category	Without category	190	47.5
	Second category	38	9.5
	First category	45	11.2
	Highest category	127	31.8

### Knowledge of AI

Among all respondents, 31.3% (*n* = 125) reported having no understanding of AI, while 68.7% (*n* = 275) indicated at least some level of familiarity. When asked specifically about the application of AI in nursing, 27.5% (*n* = 110) of the respondents stated that they had a little and 18.3% (*n* = 73) had no understanding of this aspect, suggesting that while general awareness of AI was relatively higher, knowledge of its practical use in nursing remained limited.

### Attitudes toward artificial intelligence

**Figure 1:**
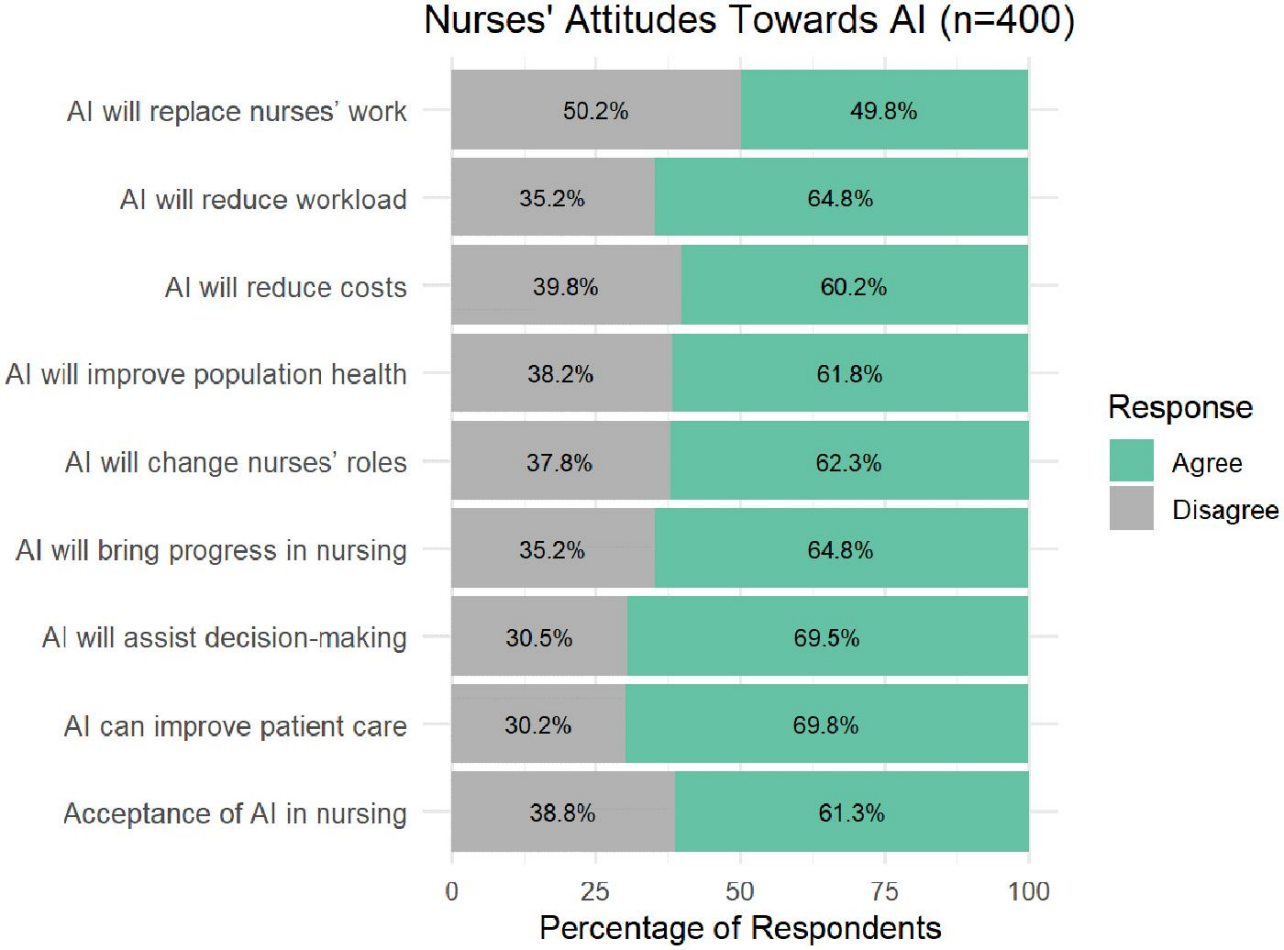
Attitudes toward AI among nurses (*n* = 400). Likert-scale responses dichotomized into binary categories.

Responses to attitude towards AI items are summarized in Figure 1. Most respondents expressed agreement (agreed or strongly agreed) that AI could have an impact in nursing practice. Specifically, over 60% of participants agreed that AI could bring progress in nursing, improve patient care, assist in decision-making, reduce workload, and influence broader aspects such as healthcare costs and population health. More than 60% (*n* = 249) also agreed that AI might change nurses’ roles, while approximately half of the respondents (49.8%, *n* = 199) considered that AI could potentially replace aspects of nursing work. Despite these perceived effects, around 38.8% (*n* = 155) expressed reluctance to personally adopt AI technologies in their practice, indicating caution in embracing AI despite recognizing its potential benefits.

Bivariate analysis revealed that younger participants were more likely to endorse AI-related items compared with older participants (*p* < 0.01– < 0.001 across items) (Table 2). No substantial differences were observed by sex, while higher professional qualification was associated with stronger endorsement of several AI items (*p* < 0.001). Place of residence showed only minor associations with perceptions of AI (*p* = 0.01–0.10).

**Table 2:** Bivariate associations between AI perceptions and sociodemographic factors (*χ*² tests).

		Age (<40 vs. ≥40)	Sex (Female vs. Male)	Prof. category (Highest vs. Lowest)	Residence (Rural vs. Urban)
№	Items	*χ*² (p)	*χ*² (p)	*χ*² (p)	*χ*² (p)
1.	AI will replace nurses’ work (Ref = Disagree)	16.24 (<0.001)	1 (0.81)	5.00 (0.03)	2.69 (0.10)
2.	AI will reduce workload (Ref = Agree)	14.42 (<0.001)	0.00 (1.00)	6.30 (0.01)	2.69 (0.10)
3.	AI will reduce costs (Ref = Agree)	9.43 (0.01)	0.77 (0.38)	8.50 (<0.001)	4.70 (0.03)
4.	AI will improve population health (Ref = Agree)	16.51 (<0.001)	0.09 (0.77)	9.34 (<0.001)	7.77 (0.01)
5.	AI will change nurses’ roles (Ref = Disagree)	13.24 (<0.001)	1 (0.81)	7.99 (<0.001)	4.91 (0.03)
6.	AI will bring progress in nursing (Ref = Agree)	12.87 (<0.001)	0.21 (0.65)	11.26 (<0.001)	5.78 (0.02)
7.	AI will assist decision making (Ref = Agree)	11.60 (<0.001)	0.29 (0.59)	13.97 (<0.001)	6.26 (0.01)
8.	AI can improve patient care (Ref = Agree)	19.25 (<0.001)	0.00 (1.00)	13.11 (<0.001)	5.42 (0.02)
9.	Acceptance of AI (Ref = Agree)	11.94 (<0.001)	0.00 (1.00)	3.37 (0.07)	5.88 (0.02)

### Factor analysis

#### Exploratory factor analysis

The overall KMO value was 0.93, indicating excellent sampling adequacy, with individual Measure of Sampling Adequacy (MSA) values ranging from 0.90 to 0.96**.** Bartlett's test of sphericity was statistically significant (*χ*^2^ = 952.05, df = 36, *p* < 0.001), confirming that the correlation matrix was appropriate for factor analysis.

Parallel analysis supported the extraction of two underlying factors, which together accounted for 72.7% of the total variance. The first factor explained 41.8% of the variance and comprised items related to the perceived impact of AI on healthcare costs, workload, nursing roles, and general acceptance of AI in practice. This factor was therefore labelled “Operational and Workforce Impact.” The second factor accounted for 30.9% of the variance and included items reflecting AI's perceived contribution to progress in nursing, clinical decision-making, patient care, and population health outcomes. Accordingly, this factor was labelled “Clinical Benefits.” Factor loadings ranged from 0.58 to 0.99, with all items loading strongly on their respective factors (Table 3). The two factors were highly correlated (r = 0.87), indicating that although conceptually distinct, they represent closely related aspects of attitudes toward AI in nursing.

**Table 3:** Standardized factor loadings of nurses’ attitudes toward AI items (*n*=400).

№	Items	EFA Loading (*n*=240)	CFA Loading (*n*=160)
Operational and Workforce Impact			
1.	Do you agree that the application of artificial intelligence in nursing will reduce healthcare costs?	0.67	0.87
2.	Do you agree that that the application of artificial intelligence will reduce the burden on healthcare workers?	0.94	0.85
3.	Do you agree that artificial intelligence in nursing will change the role of nurses in the future?	−0.86	−0.85
4.	Do you agree that artificial intelligence in nursing will replace the work of nurses?	−0.76	−0.78
5.	Do you accept the application of artificial intelligence in nursing?	0.83	0.83
Clinical and Population Health Benefits			
6.	Do you agree that artificial intelligence will revolutionize the field of nursing?	0.63	0.74
7.	Do you agree that the application of artificial intelligence in nursing can improve nursing decision-making?	0.72	0.91
8.	Do you agree that the application of artificial intelligence in nursing can improve patient care?	0.98	0.82
9.	Do you agree that artificial intelligence in nursing can improve the health of population?	0.72	0.81

### Confirmatory factor analysis

The CFA conducted on an independent subsample (*n* = 160) hypothesized two latent constructs including:

1. Clinical Benefits of AI - encompassing four items related to AI's perceived contribution to progress in nursing, enhancement of clinical decision-making, improvement of patient care, and advancement of population health outcomes.
2. Operational and Workforce Impact of AI - comprising five items reflecting perceptions of AI's potential to reduce healthcare costs and nurses’ workload, alter nursing roles, replace aspects of nursing work, and promote overall acceptance of AI in professional practice.

**Figure 2:**
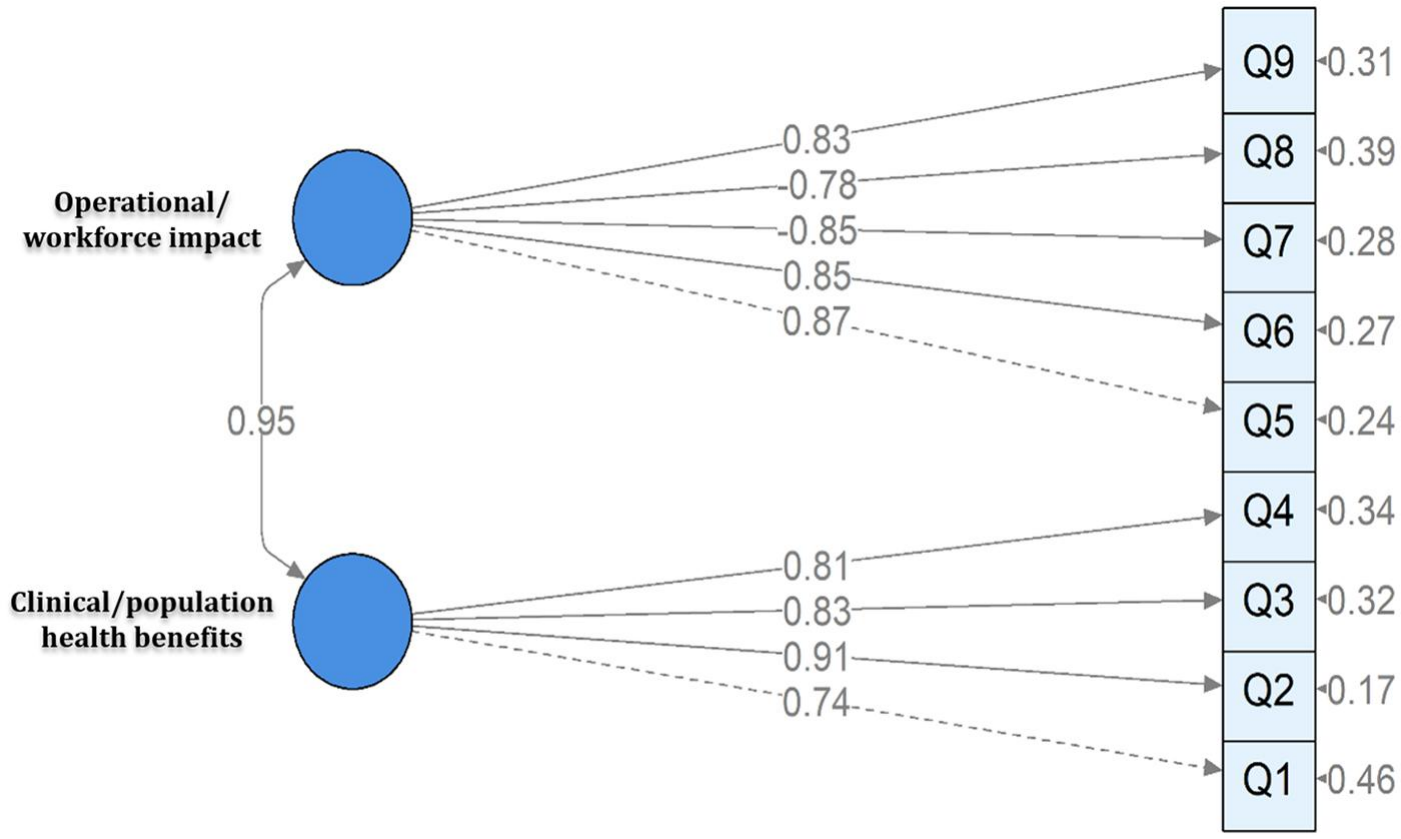
CFA path diagram of nurses’ attitudes toward AI (*n* = 160).

The CFA demonstrated a good model fit: *χ*^2^ = 37.34, *p* = 0.07; CFI=0.978; TLI=0.970; RMSEA=0.052 (90% CI: 0.021–0.077, *P* = 0.422); SRMR=0.032. All standardized factor loadings were statistically significant (*p* < 0.001), ranging from 0.73 to 0.91 for the Clinical Benefits items, and from 0.78 to 0.87 for the Operational and Workforce Impact items, demonstrating strong relationships between observed variables and their latent constructs (Table 2). In both the EFA and CFA models, the two negatively worded items (“Do you agree that artificial intelligence in nursing will change the role of nurses in the future?” and “Do you agree that artificial intelligence in nursing will replace the work of nurses?”) exhibited negative factor loadings, consistent with their reverse conceptual direction. All other positively worded items showed positive loadings.The two latent factors were highly correlated (r = 0.95), suggesting that while conceptually distinct, they represent interrelated dimensions of nurses’ overall attitudes toward AI (Figure 2). To further examine whether attitudes toward AI could be represented as a unidimensional construct, an alternative one-factor CFA model was tested. Although the one-factor model demonstrated acceptable fit indices (robust CFI=0.972; TLI=0.963; SRMR=0.033), its robust RMSEA was higher (0.087) and overall fit was inferior compared to the two-factor model. Therefore, the two-factor solution was retained due to its superior fit and stronger theoretical interpretability.

The CFA confirmed the factor structure with an excellent internal consistency (*α*=0.94). Subscale reliability was also high, with Operational and Workforce Impact (*α*=0.92) and Clinical Benefits (*α*=0.89) showing strong consistency. The average inter-item correlation was 0.68, indicating substantial positive associations among items. The CR and AVE values exceeded recommended thresholds (CR > 0.70; AVE > 0.50). Specifically, the Operational and Workforce Impact factor had CR = 0.92 and AVE=0.70, while the Clinical Benefits factor had CR = 0.89 and AVE=0.67, supporting adequate reliability and confirming that each construct accounted for a substantial proportion of the variance in its indicators (Table 4). Discriminant validity was further assessed using the Fornell–Larcker criterion (Table 4). Although the inter-factor correlation was high (r = 0.95), the two-factor model demonstrated superior fit compared with the alternative one-factor model, supporting the retention of analytically distinct but closely related constructs.

**Table 4:** Reliability, convergent and discriminant validity of the AI attitude scale.

№	Construct	Cronbach's *α*	CR	AVE	Operational and Workforce Impact	Clinical Benefits
1.	Operational and Workforce Impact	0.92	0.92	0.7	**0**.**84**	
2.	Clinical Benefits	0.89	0.89	0.67	0.95	**0**.**82**

CR, Composite Reliability; AVE, Average Variance Extracted. Diagonal elements (bold) represent the square root of AVE. Off-diagonal elements represent inter-construct correlations.

## Discussion

This study aimed to examine the psychometric properties of a scale measuring nurses’ attitudes toward AI and to explore these attitudes among PHC nurses in Almaty, Kazakhstan. Our findings indicate that while general awareness of AI was relatively high, specific knowledge about its application in nursing remained limited. Approximately one-third of respondents reported no understanding of AI, and nearly half indicated little to no understanding of AI application in nursing practice. Furthermore, a substantial proportion (38.8%) reported reluctance to personally adopt AI technologies in their practice, highlighting a cautious stance despite recognizing potential benefits. Despite this, most participants expressed positive perceptions of AI's potential to improve nursing practice, including enhancing patient care, clinical decision-making, and influencing broader operational aspects such as healthcare costs. Younger participants and those with higher professional qualifications were more likely to endorse AI items, while sex and place of residence had minimal impact. However, these findings should be interpreted cautiously, as the analyses reflect associations rather than causal relationships, and further analytical studies are needed to explore potential causal pathways.

The questionnaire demonstrated strong psychometric properties, reflecting robust construct validity and internal consistency. Factor analysis supported a two-factor structure of attitudes toward AI, comprising “Clinical Benefits” and “Operational and Workforce Impact,” which aligns with theoretical expectations and the findings of the original validation study (21). Notably, the two-factor solution in our EFA accounted for 72.7% of the total variance, which is somewhat higher than the variance explained in the original study (65.8%). This structure was subsequently confirmed through CFA in an independent sample, which also showed excellent internal consistency (Cronbach's *α* = 0.94).

The two latent factors were strongly correlated, indicating that they represent closely related dimensions of nurses’ overall attitudes toward AI. This correlation may suggest that nurses tend to evaluate AI as an integrated innovation, in which perceived improvements in patient care and clinical decision-making are closely linked to anticipated effects on workload and efficiency. This interpretation is consistent with empirical evidence showing that performance expectancy, effort expectancy, facilitating conditions, and trust jointly shape healthcare professionals’ perceptions of AI adaptation in healthcare (26, 27).

However, although nurses may experience these dimensions as part of a single overall judgment about AI, analytically distinguishing those helps clarify whether attitudes are driven primarily by anticipated clinical value or by concerns related to implementation and professional practice. In our study, factor analysis demonstrated that the items remained analytically distinguishable, supporting a two-factor structure. Specifically, the “Clinical Benefits” factor captured perceptions related mainly to patient outcomes and clinical decision support, whereas “Operational and Workforce Impact” reflects expectations regarding efficiency, workload, and professional practice. Retaining this two-factor structure therefore allows for a more nuanced understanding of nurses’ attitudes toward AI.

Our results align with global evidence indicating generally positive attitudes toward AI in healthcare. Existing literature on perceptions of nurses of AI found that predictive analytics and robotic automation are viewed positively for improving workflow and patient outcomes, but concerns remain regarding data privacy and potential deskilling across countries (27–35). Recent multicenter evidence further supports this dual perspective, demonstrating that while nurses recognize AI's clinical benefits, uncertainties related to professional autonomy, role transformation, and implementation challenges continue to shape attitudes toward adoption (34). Concerns regarding cost and the impact on empathy in patient counselling were also highlighted in earlier studies (36). On the other hand, positive attitudes toward AI were associated with higher self-efficacy and an improved subjective well-being in earlier studies (37). AI competency was shown to mediate this relationship, indicating that greater knowledge and skills in AI enhance confidence, which in turn promotes more favorable attitudes among healthcare workers (38)**.** In our study, almost half of the respondents reported having little to no understanding of AI use in nursing. This is particularly noteworthy given that a half of the participants were relatively young, a group often assumed to be more digitally literate and receptive to emerging technologies.

Research on nurses’ attitudes toward AI in Kazakhstan remains limited, with latest studies focusing on medical students rather than practicing nurses. While these student-focused studies provide insight into future workforce readiness, they are limited to capture the real-world perspectives of frontline healthcare professionals. Furthermore, existing research in the Kazakhstani context often relies on descriptive designs and rarely evaluates the psychometric properties of instruments used to measure AI attitudes, such as reliability or factor structure.

Nevertheless, a study conducted at Nazarbayev University School of Medicine found that students had limited knowledge of AI and mixed attitudes, highlighting the importance of educational interventions to improve AI readiness (39). AI integration can enhance interactive learning and prepare students for future roles, yet challenges remain in implementing AI effectively. Specific challenges are highlighted in another recent qualitative study in Astana where participants reported concerns about cybersecurity and patient data protection, indicating the need for ethical guidelines and safeguards in AI implementation (40). In Kazakhstan, concerns about cybersecurity and the protection of patient data are particularly salient. Legislative and regulatory gaps exacerbate these risks and the data protection laws only came into effect in 2024, introducing mandatory breach notification and defining “personal data security breaches” as a legal concept (41). These systemic vulnerabilities likely contribute to the caution observed in our sample, where nearly one-third of nurses were hesitant to accept AI in their practice, suggesting that real-world data security concerns may shape attitudes more than just abstract ethical debates.

This study has several strengths. First, it provides the first psychometrically validated assessment of nurses’ attitudes toward AI in Kazakh and Russian languages, using both exploratory and confirmatory factor analyses with excellent reliability and validity metrics. Second, the study included a reasonably adequate sample of practicing nurses from PHCs, offering insights into real-world perceptions. Third, the use of a two-factor model allows for a nuanced understanding of both clinical and operational aspects of AI attitudes. However, the study also has limitations. The use of self-reported measures may be subject to social desirability bias, specifically regarding knowledge on AI in healthcare and in nursing. Additionally, as the sample was limited to primary healthcare nurses in Almaty, the findings may have limited generalizability to other regions or healthcare settings. Furthermore, although the questionnaire was reviewed by a practicing nurse during adaptation, the lack of formal pilot testing may have limited the assessment of item performance prior to large-scale administration.

In conclusion, this study provides the first psychometrically validated assessment of attitudes toward AI among nurses in Kazakh and Russian languages. Nurses generally recognize the potential benefits of AI for clinical practice and operational efficiency, yet practical knowledge remains limited, and some caution persists regarding adoption. The validated scale can serve as a valuable tool for future research and for designing educational programs that enhance AI literacy, facilitate adoption, and support the ethical and optimized use of AI in nursing practice. Future studies are needed to better understand the sources of reluctance toward AI use, including the limited knowledge of AI applications in nursing observed in our sample, and to inform targeted training strategies, curriculum development, and policy initiatives.

## Data Availability

The raw data supporting the conclusions of this article will be made available by the authors, without undue reservation.
