# Supplementary material for: Assessing nurses’ attitudes toward artificial intelligence in Kazakhstan: psychometric validation of a nine-item scale
Source: Front Digit Health. 2026 Apr 1;8:1756338. doi: 10.3389/fdgth.2026.1756338 (PMC13081779; doi:10.3389/fdgth.2026.1756338)
Supplement: Supplementary file 1 [file Table1.docx]

**Sociodemogrpahic items**

1. Your gender:
   1. Female
   2. Male
2. Your age:
   1. Under 20 years
   2. 20–39 years
   3. 40–59 years
   4. 60 years and above
3. Your place of residence:
   1. Urban
   2. Rural
4. Your education level:
   1. Vocational/College
   2. Applied Bachelor
   3. Academic Bachelor
   4. Master’s degree
   5. Doctorate
5. Your professional category:
   1. None
   2. Second
   3. First
   4. Highest
6. Do you have any general awareness of artificial intelligence?
   1. Yes
   2. No
7. Do you have awareness of the application of artificial intelligence in nursing?
   1. Fully aware
   2. Mostly aware
   3. Slightly aware
   4. Not aware at all

**Attitudes towards AI items**

1. Do you agree that artificial intelligence will revolutionize the field of nursing?
   1. Strongly agree
   2. Agree
   3. Disagree
   4. Strongly disagree
2. Do you agree that the application of artificial intelligence in nursing can improve patient care?
   1. Strongly agree
   2. Agree
   3. Disagree
   4. Strongly disagree
3. Do you agree that the application of artificial intelligence in nursing can improve nursing decision-making?
   1. Strongly agree
   2. Agree
   3. Disagree
   4. Strongly disagree
4. Do you agree that artificial intelligence in nursing can improve health of population?
   1. Strongly agree
   2. Agree
   3. Disagree
   4. Strongly disagree
5. Do you agree that the application of artificial intelligence in nursing will reduce healthcare costs?
   1. Strongly agree
   2. Agree
   3. Disagree
   4. Strongly disagree
6. Do you agree that the application of artificial intelligence will reduce the burden on healthcare workers?
   1. Strongly agree
   2. Agree
   3. Disagree
   4. Strongly disagree
7. Do you agree that artificial intelligence in nursing will change the role of nurses in the future?
   1. Strongly agree
   2. Agree
   3. Disagree
   4. Strongly disagree
8. Do you agree that artificial intelligence in nursing will replace the work of nurses?
   1. Strongly agree
   2. Agree
   3. Disagree
   4. Strongly disagree
9. Do you accept the application of artificial intelligence in nursing?
   1. Strongly agree
   2. Agree
   3. Disagree
   4. Strongly disagree

**Социодемографические данные**

1. Ваш пол:

1. Женшина
2. Мужчина

2.Ваш возраст

1. Младше 20 лет
2. От 20 до 39 лет
3. От 40 до 59 лет
4. 60 лет и старше

3.Ваше место жительства:

1. Город
2. Село

4. Ваш уровень образования:

1. ТиПО (уровень колледжа)
2. Прикладной бакалавр
3. Академический бакалавр
4. Магистратура
5. Докторантура

5. Ваша категория:

1. Без категории
2. Вторая
3. Первая
4. Высшая

6. Имеете ли вы представление об искусственном интеллекте?

1. Да
2. Нет

7. Имеете ли вы представление о применении искусственного интеллекта в сестринском деле?

1. Полностью
2. Почти
3. Немного
4. Совсем не понимаю

**Отношение к ИИ**

1. Согласны ли вы с тем, что искусственный интеллект произведет прогресс в сфере сестринского дела?

1. Полностью согласен
2. Согласен
3. Не согласен
4. Полностью не согласен

2. Согласны ли вы с тем, что применение искусственного интеллекта в сестринском деле может улучшить уход за пациентами?

1. Полностью согласен
2. Согласен
3. Не согласен
4. Полностью не согласен

3. Согласны ли вы с тем, что применение искусственного интеллекта в сестринском деле может улучшить процесс принятия решений медсестрами?

1. Полностью согласен
2. Согласен
3. Не согласен
4. Полностью не согласен

4. Согласны ли вы с тем, что искусственный интеллект в сестринском деле может улучшить здоровье населения?

1. Полностью согласен
2. Согласен
3. Не согласен
4. Полностью не согласен

5. Согласны ли вы с тем, что применение искусственного интеллекта в сестринском деле снизит затраты на здравоохранение?

1. Полностью согласен
2. Согласен
3. Не согласен
4. Полностью не согласен

6. Согласны ли вы с тем, что применение искусственного интеллекта снизит нагрузку на медицинских работников?

1. Полностью согласен
2. Согласен
3. Не согласен
4. Полностью не согласен

7. Согласны ли вы с тем, что искусственный интеллект в сестринском деле изменит роль медсестер в будущем?

1. Полностью согласен
2. Согласен
3. Не согласен
4. Полностью не согласен

8. Согласны ли вы с тем, что искусственный интеллект в сестринском деле заменит работу медсестер?

1. Полностью согласен
2. Согласен
3. Не согласен
4. Полностью не согласен

9. Принимаете ли вы применение искусственного интеллекта в сестринском деле?

1. Полностью согласен
2. Согласен
3. Не согласен
4. Полностью не согласен

**Әлеуметтік–демографиялық деректер**

1. Сіздің жынысыңыз:

1. Әйел
2. Ер

2. Сіздің жасыңыз:

1. 20 жасқа дейін
2. 20-39 жас аралығы
3. 40-59 жас аралығы
4. 60 жастан жоғары

3. Сіздің тұрғылықты жеріңіз?

1. Қала
2. Ауыл

4. Сіздің білім деңгейіңіз:

1. Техникалық және кәсіптік білім (колледж деңгейі)
2. Қолданбалы бакалавриат
3. Академиялық бакалавриат
4. Магистратура
5. Докторантура

5. Сіздің категорияңыз:

1. Категориясыз
2. Екінші
3. Бірінші
4. Жоғары

6. Сізде жасанды интеллект туралы түсінік бар ма?

1. Иә
2. Жоқ
3. Мүлдем түсінігім жоқ

### 7. Сізде мейіргер ісінде жасанды интеллектіні қолдану жөнінде түсінік бар ма?

1. Толықтай
2. Шамамен
3. Аздап
4. Мүлде түсінбеймін

**ЖИ-ға деген көзқарас**

1. Сіз жасанды интеллект мейіргер ісінде өзгеріс жасайды деп ойлайсыз ба?

1. Толық келісемін
2. Келісемін
3. Келіспеймін
4. Мүлдем келіспеймін

2. Сіздің ойыңызша, жасанды интеллект мейіргер ісінде пациенттерге күтімді жақсарта ала ма?

1. Толық келісемін
2. Келісемін
3. Келіспеймін
4. Мүлдем келіспеймін

3. Сіздің ойыңызша, жасанды интеллект мейіргерлердің шешім қабылдауына ықпал етеді ме?

1. Толық келісемін
2. Келісемін
3. Келіспеймін
4. Мүлдем келіспеймін

4. Жасанды интеллект халық денсаулығын жақсарта алады деп ойлайсыз ба?

1. Толық келісемін
2. Келісемін
3. Келіспеймін
4. Мүлдем келіспеймін

5. Сіздің ойыңызша, жасанды интеллект денсаулық сақтау шығындарын азайтуға көмектеседі ме?

1. Толық келісемін
2. Келісемін
3. Келіспеймін
4. Мүлдем келіспеймін

6. Сіздің ойыңызша, жасанды интеллект медициналық қызметкерлердің жұмыс жүктемесін азайтуға көмектеседі ме?

1. Толық келісемін
2. Келісемін
3. Келіспеймін
4. Мүлдем келіспеймін

7. Сіздің ойыңызша жасанды интеллект болашақта мейіргерлердің рөлін өзгертеді деп ойлайсыз ба?

1. Толық келісемін
2. Келісемін
3. Келіспеймін
4. Мүлдем келіспеймін

8. Сіздің ойыңызша жасанды интеллект мейіргерлердің жұмысын алмастыра алады деп ойлайсыз ба?

1. Толық келісемін
2. Келісемін
3. Келіспеймін
4. Мүлдем келіспеймін

9. Сіз мейіргер ісінде жасанды интеллектті қолдануды қолдайсыз ба?

1. Толық келісемін
2. Келісемін
3. Келіспеймін
4. Мүлдем келіспеймін
